# 1,3-Bis(1-benzyl-1*H*-benzimidazol-2-yl)-2-oxapropane

**DOI:** 10.1107/S1600536809012781

**Published:** 2009-04-10

**Authors:** Huilu Wu, Ruirui Yun, Kaitong Wang, Xingcai Huang, Qingyu Sun

**Affiliations:** a School of Chemical and Biological Engineering, Lanzhou Jiaotong University, Lanzhou 730070, People’s Republic of China

## Abstract

In the title compound, C_30_H_26_N_4_O, the dihedral angle between the two benzimidazole rings is 69.35 (9)°. The dihedral angles between the benzimidazole ring system and the phenyl ring are 76.79 (12) and 86.10 (11)° in the two benzyl­benzimidazole moieties.

## Related literature

For the biological activity of the benzimidazole core, see: Horton *et al.* (2003 ▶). For the anti­protozoal activity of 2- and 5-substituted benzimidazoles, see: Navarrete-Vázquez *et al.* (2001 ▶).
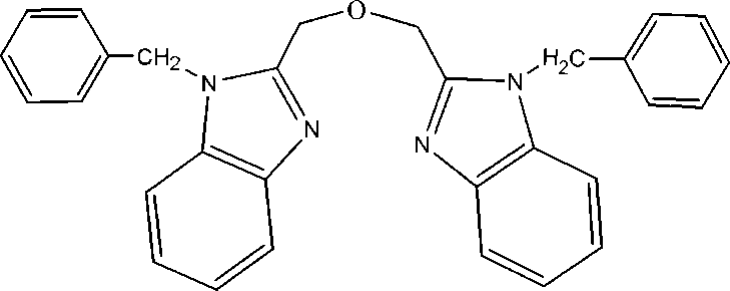


## Experimental

### 

#### Crystal data

C_30_H_26_N_4_O
*M* *_r_* = 458.55
Triclinic, 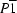

*a* = 8.5477 (3) Å
*b* = 11.8976 (5) Å
*c* = 12.3961 (5) Å
α = 101.300 (1)°
β = 92.394 (1)°
γ = 107.765 (1)°
*V* = 1170.28 (8) Å^3^
*Z* = 2
Mo *K*α radiation
μ = 0.08 mm^−1^
*T* = 153 K
0.58 × 0.52 × 0.19 mm

#### Data collection

Rigaku R-AXIS Spider diffractometer
Absorption correction: multi-scan (*ABSCOR*; Higashi, 1995 ▶) *T*_min_ = 0.955, *T*_max_ = 0.985
11531 measured reflections
5275 independent reflections
4542 reflections with *I* > 2σ(*I*)
*R*_int_ = 0.013

#### Refinement

*R*[*F* ^2^ > 2σ(*F* ^2^)] = 0.038
*wR*(*F* ^2^) = 0.126
*S* = 1.09
5275 reflections
317 parameters
H-atom parameters constrained
Δρ_max_ = 0.50 e Å^−3^
Δρ_min_ = −0.34 e Å^−3^

### 

Data collection: *RAPID-AUTO* (Rigaku/MSC, 2004 ▶); cell refinement: *RAPID-AUTO*; data reduction: *RAPID-AUTO*; program(s) used to solve structure: *SHELXS97* (Sheldrick, 2008 ▶); program(s) used to refine structure: *SHELXL97* (Sheldrick, 2008 ▶); molecular graphics: *SHELXTL* (Sheldrick, 2008 ▶); software used to prepare material for publication: *SHELXTL*.

## Supplementary Material

Crystal structure: contains datablocks global, I. DOI: 10.1107/S1600536809012781/lh2800sup1.cif

Structure factors: contains datablocks I. DOI: 10.1107/S1600536809012781/lh2800Isup2.hkl

Additional supplementary materials:  crystallographic information; 3D view; checkCIF report

## Figures and Tables

**Fig. 1.**
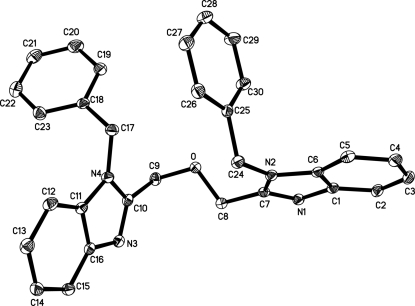
Molecular structure of the title compound. Hydrogen atoms have been omitted for clarity and the displacement ellipsoids are shown at the 30% probability level.

## supplementary crystallographic information

### Comment

Benzimidazole derivatives, such as mebendazole and albendazole, are used as anthelmintic drugs. More recently, the antiprotozoal activity of 2- and 5-substituted benzimidazoles has been reported (Navarrete-Vázquez *et al.* 2001). The benzimidazole core is of interest because of its diverse biological activities, and it is a well known structure in medicinal chemistry (Horton *et al.* 2003). The molecular structure of the title compound is shown in Fig. 1. The dihedral angle between N3/N4/C10-C16 and C18-C23 is 76.79 (12)° and that between N1/N2/C1-C7 and C25-C30 is 86.10 (11)°.

### Experimental

A solution of 5.56 (20 mmol) of 1,3-bis(benzimidazol-2-yl)-2-oxopropane with 1.56 g (40 mmol) potassium in 150 ml tetrahydrofuran followed by addition of 5.06 g (40 mmol) benzyl bromide was concentrated and recrystallized from methanol, formimg white block crystals suitable for X-ray diffraction studies. (found: C, 78.51; H, 5.73; N,12.24 Calcd. for C_30_H_26_N_4_O: C, 78.58; H, 5.71; N, 12.22)

### Refinement

All H atoms were positioned geometrically with C—H distances ranging from 0.95 to 0.99 Å and allowed to ride on their parent atoms with *U*_iso_(H) = 1.2 *U*_eq_ of the carrier atom.

### Crystal data

**Table:**

C_30_H_26_N_4_O	*Z* = 2
*M**_r_* = 458.55	*F*(000) = 484
Triclinic, *P*1	*D*_x_ = 1.301 Mg m^−^^3^
Hall symbol: -P 1	Melting point = 450–451 K
*a* = 8.5477 (3) Å	Mo *K*α radiation, λ = 0.71073 Å
*b* = 11.8976 (5) Å	Cell parameters from 5275 reflections
*c* = 12.3961 (5) Å	θ = 3.0–27.5°
α = 101.300 (1)°	µ = 0.08 mm^−^^1^
β = 92.394 (1)°	*T* = 153 K
γ = 107.765 (1)°	Block, white
*V* = 1170.28 (8) Å^3^	0.58 × 0.52 × 0.19 mm

### Data collection

**Table:**

Rigaku R-AXIS Spider diffractometer	5275 independent reflections
Radiation source: fine-focus sealed tube	4542 reflections with *I* > 2σ(*I*)
graphite	*R*_int_ = 0.013
φ and ω scans	θ_max_ = 27.5°, θ_min_ = 3.0°
Absorption correction: multi-scan (*ABSCOR*; Higashi, 1995)	*h* = −11→10
*T*_min_ = 0.955, *T*_max_ = 0.985	*k* = −15→15
11531 measured reflections	*l* = −16→16

### Refinement

**Table:**

Refinement on *F*^2^	Secondary atom site location: difference Fourier map
Least-squares matrix: full	Hydrogen site location: inferred from neighbouring sites
*R*[*F*^2^ > 2σ(*F*^2^)] = 0.038	H-atom parameters constrained
*wR*(*F*^2^) = 0.126	*w* = 1/[σ^2^(*F*_o_^2^) + (0.0736*P*)^2^ + 0.3041*P*] where *P* = (*F*_o_^2^ + 2*F*_c_^2^)/3
*S* = 1.09	(Δ/σ)_max_ < 0.001
5275 reflections	Δρ_max_ = 0.50 e Å^−^^3^
317 parameters	Δρ_min_ = −0.34 e Å^−^^3^
0 restraints	Extinction correction: *SHELXL*, Fc^*^=kFc[1+0.001xFc^2^λ^3^/sin(2θ)]^-1/4^
Primary atom site location: structure-invariant direct methods	Extinction coefficient: 0.044 (4)

### Special details

**Table:**

Geometry. All e.s.d.'s (except the e.s.d. in the dihedral angle between two l.s. planes) are estimated using the full covariance matrix. The cell e.s.d.'s are taken into account individually in the estimation of e.s.d.'s in distances, angles and torsion angles; correlations between e.s.d.'s in cell parameters are only used when they are defined by crystal symmetry. An approximate (isotropic) treatment of cell e.s.d.'s is used for estimating e.s.d.'s involving l.s. planes.
Refinement. Refinement of *F*^2^ against ALL reflections. The weighted *R*-factor *wR* and goodness of fit *S* are based on *F*^2^, conventional *R*-factors *R* are based on *F*, with *F* set to zero for negative *F*^2^. The threshold expression of *F*^2^ > σ(*F*^2^) is used only for calculating *R*-factors(gt) *etc*. and is not relevant to the choice of reflections for refinement. *R*-factors based on *F*^2^ are statistically about twice as large as those based on *F*, and *R*- factors based on ALL data will be even larger.

### Fractional atomic coordinates and isotropic or equivalent isotropic displacement parameters (Å^2^)

**Table:**

	*x*	*y*	*z*	*U*_iso_*/*U*_eq_	
O	0.80479 (11)	0.49510 (8)	0.70677 (7)	0.0252 (2)	
N1	0.73219 (13)	0.71312 (9)	0.90708 (8)	0.0233 (2)	
N2	0.54296 (12)	0.52783 (9)	0.85764 (8)	0.0214 (2)	
N3	0.99857 (12)	0.31236 (9)	0.79071 (8)	0.0231 (2)	
N4	0.77779 (12)	0.22141 (9)	0.66368 (8)	0.0231 (2)	
C1	0.58038 (15)	0.71864 (10)	0.93941 (9)	0.0213 (2)	
C2	0.53783 (16)	0.81685 (11)	0.99680 (10)	0.0256 (3)	
H2	0.6172	0.8953	1.0182	0.031*	
C3	0.37662 (17)	0.79594 (12)	1.02133 (10)	0.0276 (3)	
H3	0.3451	0.8610	1.0611	0.033*	
C4	0.25816 (16)	0.68035 (12)	0.98864 (10)	0.0276 (3)	
H4	0.1483	0.6695	1.0065	0.033*	
C5	0.29667 (15)	0.58181 (11)	0.93114 (10)	0.0247 (3)	
H5	0.2164	0.5039	0.9086	0.030*	
C6	0.46015 (15)	0.60377 (10)	0.90831 (9)	0.0208 (2)	
C7	0.70389 (15)	0.59876 (11)	0.85997 (9)	0.0217 (2)	
C8	0.83822 (16)	0.55036 (12)	0.82143 (10)	0.0264 (3)	
H8A	0.9446	0.6171	0.8349	0.032*	
H8B	0.8481	0.4902	0.8640	0.032*	
C9	0.92732 (15)	0.44005 (11)	0.67180 (10)	0.0258 (3)	
H9A	1.0385	0.4975	0.7013	0.031*	
H9B	0.9208	0.4227	0.5900	0.031*	
C10	0.90483 (14)	0.32529 (11)	0.71040 (10)	0.0222 (2)	
C11	0.79369 (14)	0.13374 (11)	0.71787 (9)	0.0221 (2)	
C12	0.70094 (16)	0.01242 (12)	0.70590 (11)	0.0284 (3)	
H12	0.6078	−0.0257	0.6520	0.034*	
C13	0.75138 (17)	−0.04945 (12)	0.77646 (11)	0.0295 (3)	
H13	0.6919	−0.1325	0.7706	0.035*	
C14	0.88820 (17)	0.00726 (12)	0.85663 (11)	0.0283 (3)	
H14	0.9187	−0.0382	0.9040	0.034*	
C15	0.98002 (15)	0.12805 (12)	0.86844 (10)	0.0258 (3)	
H15	1.0722	0.1661	0.9231	0.031*	
C16	0.93194 (14)	0.19187 (10)	0.79690 (10)	0.0212 (2)	
C17	0.64947 (15)	0.20654 (12)	0.57568 (10)	0.0257 (3)	
H17A	0.6159	0.2803	0.5873	0.031*	
H17B	0.5516	0.1378	0.5808	0.031*	
C18	0.70267 (15)	0.18411 (11)	0.46087 (10)	0.0236 (3)	
C19	0.66731 (18)	0.24654 (12)	0.38477 (11)	0.0312 (3)	
H19	0.6115	0.3040	0.4057	0.037*	
C20	0.7125 (2)	0.22588 (14)	0.27833 (12)	0.0403 (4)	
H20	0.6869	0.2686	0.2266	0.048*	
C21	0.7950 (2)	0.14290 (14)	0.24755 (12)	0.0393 (3)	
H21	0.8275	0.1294	0.1750	0.047*	
C22	0.82983 (19)	0.07973 (13)	0.32288 (12)	0.0363 (3)	
H22	0.8861	0.0225	0.3020	0.044*	
C23	0.78271 (17)	0.09980 (12)	0.42866 (11)	0.0303 (3)	
H23	0.8055	0.0553	0.4797	0.036*	
C24	0.46863 (16)	0.39871 (10)	0.81196 (10)	0.0250 (3)	
H24A	0.3901	0.3635	0.8622	0.030*	
H24B	0.5565	0.3603	0.8095	0.030*	
C25	0.37816 (14)	0.36798 (10)	0.69711 (10)	0.0214 (2)	
C26	0.28424 (15)	0.24803 (12)	0.65272 (12)	0.0289 (3)	
H26	0.2773	0.1885	0.6950	0.035*	
C27	0.20114 (18)	0.21494 (13)	0.54754 (13)	0.0383 (3)	
H27	0.1383	0.1327	0.5179	0.046*	
C28	0.20876 (18)	0.30063 (14)	0.48511 (12)	0.0382 (3)	
H28	0.1505	0.2778	0.4132	0.046*	
C29	0.30168 (19)	0.41940 (14)	0.52837 (12)	0.0352 (3)	
H29	0.3079	0.4787	0.4859	0.042*	
C30	0.38665 (17)	0.45315 (12)	0.63410 (11)	0.0287 (3)	
H30	0.4508	0.5352	0.6631	0.034*	

### Atomic displacement parameters (Å^2^)

**Table:**

	*U*^11^	*U*^22^	*U*^33^	*U*^12^	*U*^13^	*U*^23^
O	0.0294 (4)	0.0282 (4)	0.0204 (4)	0.0137 (4)	0.0023 (3)	0.0041 (3)
N1	0.0256 (5)	0.0211 (5)	0.0220 (5)	0.0072 (4)	−0.0003 (4)	0.0032 (4)
N2	0.0266 (5)	0.0177 (5)	0.0187 (5)	0.0064 (4)	−0.0011 (4)	0.0031 (4)
N3	0.0228 (5)	0.0223 (5)	0.0224 (5)	0.0066 (4)	0.0023 (4)	0.0019 (4)
N4	0.0236 (5)	0.0234 (5)	0.0208 (5)	0.0060 (4)	−0.0005 (4)	0.0043 (4)
C1	0.0254 (6)	0.0206 (5)	0.0176 (5)	0.0066 (4)	0.0005 (4)	0.0051 (4)
C2	0.0331 (6)	0.0207 (6)	0.0217 (6)	0.0085 (5)	0.0023 (5)	0.0023 (4)
C3	0.0374 (7)	0.0288 (6)	0.0200 (6)	0.0159 (5)	0.0062 (5)	0.0044 (5)
C4	0.0297 (6)	0.0346 (7)	0.0204 (6)	0.0120 (5)	0.0047 (5)	0.0075 (5)
C5	0.0265 (6)	0.0262 (6)	0.0192 (5)	0.0046 (5)	0.0011 (4)	0.0060 (4)
C6	0.0268 (6)	0.0208 (5)	0.0147 (5)	0.0072 (4)	0.0002 (4)	0.0045 (4)
C7	0.0250 (6)	0.0221 (6)	0.0181 (5)	0.0079 (4)	−0.0011 (4)	0.0046 (4)
C8	0.0286 (6)	0.0284 (6)	0.0225 (6)	0.0128 (5)	−0.0019 (5)	0.0015 (5)
C9	0.0272 (6)	0.0257 (6)	0.0260 (6)	0.0094 (5)	0.0086 (5)	0.0065 (5)
C10	0.0217 (5)	0.0223 (6)	0.0217 (5)	0.0071 (4)	0.0051 (4)	0.0021 (4)
C11	0.0240 (6)	0.0232 (6)	0.0182 (5)	0.0071 (5)	0.0030 (4)	0.0034 (4)
C12	0.0294 (6)	0.0251 (6)	0.0243 (6)	0.0019 (5)	−0.0017 (5)	0.0022 (5)
C13	0.0358 (7)	0.0217 (6)	0.0281 (6)	0.0046 (5)	0.0050 (5)	0.0056 (5)
C14	0.0343 (7)	0.0292 (6)	0.0253 (6)	0.0138 (5)	0.0050 (5)	0.0083 (5)
C15	0.0256 (6)	0.0290 (6)	0.0224 (6)	0.0099 (5)	0.0003 (5)	0.0036 (5)
C16	0.0209 (5)	0.0211 (5)	0.0201 (5)	0.0064 (4)	0.0040 (4)	0.0011 (4)
C17	0.0225 (6)	0.0314 (6)	0.0229 (6)	0.0092 (5)	0.0002 (5)	0.0048 (5)
C18	0.0225 (6)	0.0210 (5)	0.0230 (6)	0.0025 (4)	−0.0037 (4)	0.0030 (4)
C19	0.0393 (7)	0.0268 (6)	0.0285 (6)	0.0122 (5)	−0.0017 (5)	0.0070 (5)
C20	0.0543 (9)	0.0395 (8)	0.0271 (7)	0.0124 (7)	−0.0023 (6)	0.0127 (6)
C21	0.0470 (8)	0.0395 (8)	0.0243 (6)	0.0057 (6)	0.0046 (6)	0.0036 (6)
C22	0.0408 (8)	0.0321 (7)	0.0352 (7)	0.0125 (6)	0.0092 (6)	0.0032 (6)
C23	0.0348 (7)	0.0297 (6)	0.0298 (7)	0.0132 (5)	0.0044 (5)	0.0096 (5)
C24	0.0343 (6)	0.0171 (5)	0.0223 (6)	0.0064 (5)	0.0001 (5)	0.0048 (4)
C25	0.0205 (5)	0.0211 (6)	0.0217 (6)	0.0071 (4)	0.0030 (4)	0.0019 (4)
C26	0.0247 (6)	0.0222 (6)	0.0367 (7)	0.0056 (5)	0.0011 (5)	0.0027 (5)
C27	0.0330 (7)	0.0272 (7)	0.0438 (8)	0.0053 (5)	−0.0093 (6)	−0.0076 (6)
C28	0.0369 (7)	0.0422 (8)	0.0285 (7)	0.0121 (6)	−0.0081 (6)	−0.0050 (6)
C29	0.0403 (7)	0.0370 (7)	0.0262 (7)	0.0099 (6)	−0.0040 (6)	0.0077 (6)
C30	0.0337 (7)	0.0238 (6)	0.0245 (6)	0.0044 (5)	−0.0022 (5)	0.0046 (5)

### Geometric parameters (Å, °)

**Table:**

O—C8	1.4211 (14)	C13—H13	0.9500
O—C9	1.4350 (14)	C14—C15	1.3865 (18)
N1—C7	1.3141 (15)	C14—H14	0.9500
N1—C1	1.3907 (16)	C15—C16	1.3985 (17)
N2—C7	1.3726 (15)	C15—H15	0.9500
N2—C6	1.3846 (15)	C17—C18	1.5108 (17)
N2—C24	1.4553 (14)	C17—H17A	0.9900
N3—C10	1.3136 (16)	C17—H17B	0.9900
N3—C16	1.3905 (15)	C18—C23	1.3850 (18)
N4—C10	1.3748 (15)	C18—C19	1.3859 (18)
N4—C11	1.3825 (15)	C19—C20	1.387 (2)
N4—C17	1.4596 (15)	C19—H19	0.9500
C1—C2	1.3996 (17)	C20—C21	1.384 (2)
C1—C6	1.4056 (16)	C20—H20	0.9500
C2—C3	1.3816 (18)	C21—C22	1.383 (2)
C2—H2	0.9500	C21—H21	0.9500
C3—C4	1.4065 (19)	C22—C23	1.385 (2)
C3—H3	0.9500	C22—H22	0.9500
C4—C5	1.3851 (18)	C23—H23	0.9500
C4—H4	0.9500	C24—C25	1.5134 (16)
C5—C6	1.3929 (17)	C24—H24A	0.9900
C5—H5	0.9500	C24—H24B	0.9900
C7—C8	1.4898 (17)	C25—C30	1.3828 (17)
C8—H8A	0.9900	C25—C26	1.3930 (16)
C8—H8B	0.9900	C26—C27	1.382 (2)
C9—C10	1.4945 (17)	C26—H26	0.9500
C9—H9A	0.9900	C27—C28	1.384 (2)
C9—H9B	0.9900	C27—H27	0.9500
C11—C12	1.3935 (17)	C28—C29	1.378 (2)
C11—C16	1.4049 (16)	C28—H28	0.9500
C12—C13	1.3811 (19)	C29—C30	1.3938 (18)
C12—H12	0.9500	C29—H29	0.9500
C13—C14	1.4033 (19)	C30—H30	0.9500
			
C8—O—C9	110.65 (9)	C13—C14—H14	119.2
C7—N1—C1	104.47 (10)	C14—C15—C16	117.55 (11)
C7—N2—C6	106.33 (9)	C14—C15—H15	121.2
C7—N2—C24	128.47 (10)	C16—C15—H15	121.2
C6—N2—C24	125.20 (10)	N3—C16—C15	130.02 (11)
C10—N3—C16	104.83 (10)	N3—C16—C11	109.99 (10)
C10—N4—C11	106.39 (10)	C15—C16—C11	119.97 (11)
C10—N4—C17	127.05 (11)	N4—C17—C18	113.67 (10)
C11—N4—C17	126.55 (10)	N4—C17—H17A	108.8
N1—C1—C2	129.86 (11)	C18—C17—H17A	108.8
N1—C1—C6	110.28 (10)	N4—C17—H17B	108.8
C2—C1—C6	119.85 (11)	C18—C17—H17B	108.8
C3—C2—C1	117.78 (11)	H17A—C17—H17B	107.7
C3—C2—H2	121.1	C23—C18—C19	118.91 (12)
C1—C2—H2	121.1	C23—C18—C17	121.38 (11)
C2—C3—C4	121.34 (12)	C19—C18—C17	119.69 (11)
C2—C3—H3	119.3	C18—C19—C20	120.65 (13)
C4—C3—H3	119.3	C18—C19—H19	119.7
C5—C4—C3	122.06 (12)	C20—C19—H19	119.7
C5—C4—H4	119.0	C21—C20—C19	119.97 (13)
C3—C4—H4	119.0	C21—C20—H20	120.0
C4—C5—C6	116.01 (11)	C19—C20—H20	120.0
C4—C5—H5	122.0	C22—C21—C20	119.69 (13)
C6—C5—H5	122.0	C22—C21—H21	120.2
N2—C6—C5	131.85 (11)	C20—C21—H21	120.2
N2—C6—C1	105.17 (10)	C21—C22—C23	120.05 (14)
C5—C6—C1	122.96 (11)	C21—C22—H22	120.0
N1—C7—N2	113.75 (11)	C23—C22—H22	120.0
N1—C7—C8	122.39 (11)	C18—C23—C22	120.71 (12)
N2—C7—C8	123.68 (11)	C18—C23—H23	119.6
O—C8—C7	110.75 (10)	C22—C23—H23	119.6
O—C8—H8A	109.5	N2—C24—C25	114.13 (10)
C7—C8—H8A	109.5	N2—C24—H24A	108.7
O—C8—H8B	109.5	C25—C24—H24A	108.7
C7—C8—H8B	109.5	N2—C24—H24B	108.7
H8A—C8—H8B	108.1	C25—C24—H24B	108.7
O—C9—C10	112.03 (10)	H24A—C24—H24B	107.6
O—C9—H9A	109.2	C30—C25—C26	118.82 (11)
C10—C9—H9A	109.2	C30—C25—C24	122.96 (11)
O—C9—H9B	109.2	C26—C25—C24	118.21 (11)
C10—C9—H9B	109.2	C27—C26—C25	120.48 (13)
H9A—C9—H9B	107.9	C27—C26—H26	119.8
N3—C10—N4	113.39 (11)	C25—C26—H26	119.8
N3—C10—C9	125.14 (11)	C26—C27—C28	120.52 (13)
N4—C10—C9	121.46 (11)	C26—C27—H27	119.7
N4—C11—C12	131.98 (11)	C28—C27—H27	119.7
N4—C11—C16	105.38 (10)	C29—C28—C27	119.31 (13)
C12—C11—C16	122.64 (11)	C29—C28—H28	120.3
C13—C12—C11	116.55 (11)	C27—C28—H28	120.3
C13—C12—H12	121.7	C28—C29—C30	120.41 (13)
C11—C12—H12	121.7	C28—C29—H29	119.8
C12—C13—C14	121.68 (12)	C30—C29—H29	119.8
C12—C13—H13	119.2	C25—C30—C29	120.44 (12)
C14—C13—H13	119.2	C25—C30—H30	119.8
C15—C14—C13	121.61 (12)	C29—C30—H30	119.8
C15—C14—H14	119.2		
			
C7—N1—C1—C2	177.64 (12)	C17—N4—C11—C16	−178.40 (11)
C7—N1—C1—C6	−0.91 (12)	N4—C11—C12—C13	−179.01 (13)
N1—C1—C2—C3	−178.07 (12)	C16—C11—C12—C13	0.11 (19)
C6—C1—C2—C3	0.35 (17)	C11—C12—C13—C14	0.5 (2)
C1—C2—C3—C4	−0.82 (18)	C12—C13—C14—C15	−0.4 (2)
C2—C3—C4—C5	0.42 (19)	C13—C14—C15—C16	−0.32 (19)
C3—C4—C5—C6	0.47 (18)	C10—N3—C16—C15	−178.95 (12)
C7—N2—C6—C5	−178.66 (12)	C10—N3—C16—C11	−0.61 (13)
C24—N2—C6—C5	1.42 (19)	C14—C15—C16—N3	179.09 (12)
C7—N2—C6—C1	−0.40 (12)	C14—C15—C16—C11	0.90 (17)
C24—N2—C6—C1	179.68 (10)	N4—C11—C16—N3	−0.03 (13)
C4—C5—C6—N2	177.04 (12)	C12—C11—C16—N3	−179.35 (11)
C4—C5—C6—C1	−0.95 (17)	N4—C11—C16—C15	178.50 (10)
N1—C1—C6—N2	0.82 (12)	C12—C11—C16—C15	−0.82 (18)
C2—C1—C6—N2	−177.89 (10)	C10—N4—C17—C18	82.90 (15)
N1—C1—C6—C5	179.27 (10)	C11—N4—C17—C18	−98.27 (14)
C2—C1—C6—C5	0.56 (17)	N4—C17—C18—C23	47.20 (16)
C1—N1—C7—N2	0.66 (13)	N4—C17—C18—C19	−134.51 (12)
C1—N1—C7—C8	−174.66 (11)	C23—C18—C19—C20	−0.6 (2)
C6—N2—C7—N1	−0.17 (13)	C17—C18—C19—C20	−178.91 (12)
C24—N2—C7—N1	179.75 (11)	C18—C19—C20—C21	−0.5 (2)
C6—N2—C7—C8	175.08 (10)	C19—C20—C21—C22	0.9 (2)
C24—N2—C7—C8	−5.00 (18)	C20—C21—C22—C23	−0.2 (2)
C9—O—C8—C7	−175.36 (10)	C19—C18—C23—C22	1.3 (2)
N1—C7—C8—O	−122.70 (12)	C17—C18—C23—C22	179.60 (12)
N2—C7—C8—O	62.45 (15)	C21—C22—C23—C18	−0.9 (2)
C8—O—C9—C10	74.56 (13)	C7—N2—C24—C25	−98.23 (14)
C16—N3—C10—N4	1.06 (13)	C6—N2—C24—C25	81.68 (14)
C16—N3—C10—C9	−179.86 (11)	N2—C24—C25—C30	9.57 (17)
C11—N4—C10—N3	−1.10 (14)	N2—C24—C25—C26	−171.39 (11)
C17—N4—C10—N3	177.92 (11)	C30—C25—C26—C27	0.00 (19)
C11—N4—C10—C9	179.78 (10)	C24—C25—C26—C27	−179.08 (12)
C17—N4—C10—C9	−1.20 (18)	C25—C26—C27—C28	−0.6 (2)
O—C9—C10—N3	−106.11 (13)	C26—C27—C28—C29	0.7 (2)
O—C9—C10—N4	72.90 (14)	C27—C28—C29—C30	−0.3 (2)
C10—N4—C11—C12	179.86 (13)	C26—C25—C30—C29	0.43 (19)
C17—N4—C11—C12	0.8 (2)	C24—C25—C30—C29	179.46 (13)
C10—N4—C11—C16	0.63 (13)	C28—C29—C30—C25	−0.3 (2)
